# Tics and Their Treatment

**Published:** 1908-09

**Authors:** 


**Tics and their Treatment.** By HENRY MEIGE and E. FERRIDEL, with a Preface by Professor BRISSAND. Translated and edited, with a critical appendix, by S. A. K. WILSON, M.A., M.D. Pp. xxi., 386. London: Sidney Appleton. 1907.

This is a most interesting book, and should be read by all who follow the progress of neurology.

We have in English no exact equivalent for the word tic: we may quote the author's definition. He defines tic as a co-ordinated purposive act, provoked in the first instance by some external cause or by an idea; repetition leads to its becoming habitual, and finally to its involuntary reproduction without cause and for no purpose. At the same time, as its form, intensity, and frequency are exaggerated, it thus assumes the characters of a convulsive movement, inopportune and excessive; its execution is often preceded by an irresistible impulse, its suppression associated with malaise. The effect of distraction or of volitional effort is to diminish its activity. In sleep it disappears. It occurs in predisposed individuals, who usually show other indications of mental instability.

Perusal of the book certainly leads us to accept the author's view that the tics are psychomotor disorders—belong, that is, to the psychoneuroses. A number of cases which support the author's contention as to the pathogeny of the tics is given in the text. Certainly this view affords a more satisfactory basis for treatment, and the chapters on the mode of treatment by re-education—that is, by regularly graduated exercises of the muscles of the area affected by tic—deserves careful study in view of the successful results attained. We can cordially recommend this book as a valuable contribution to a difficult subject. It is well translated, and the translator has enhanced its value by adding M. Meige's latest definitions and views from his monograph *Le Tics*, and also by the addition of a full bibliography.

